# Role of arachidonic acid in ischemic heart disease under different comorbidities: risk or protection?

**DOI:** 10.1186/s12933-024-02277-0

**Published:** 2024-06-24

**Authors:** Chengjia Li, Huijun Chen

**Affiliations:** 1 Heilongjiang University of Chinese Medicine, Harbin, 150000 China; 2 The second affiliated hospital, Heilongjiang University of Chinese Medicine, Harbin, 150001 China

**Keywords:** Arachidonic acid, Ferroptosis, Myocardial ischemic, Mitochondrial function

## Abstract

In a translational study involving animal models and human subjects, Lv et al. demonstrate that arachidonic acid (AA) exhibits cardioprotective effects in diabetic myocardial ischemia, suggesting a departure from its known role in promoting ferroptosis—a form of cell death characterized by iron-dependent lipid peroxidation. However, the study does not address how underlying diabetic conditions might influence the metabolic pathways of AA, which are critical for fully understanding its impact on heart disease. Diabetes can significantly alter lipid metabolism, which in turn might affect the enzymatic processes involved in AA’s metabolism, leading to different outcomes in the disease process. Further examination of the role of diabetes in modulating AA’s effects could enhance the understanding of its protective mechanism in ischemic conditions. This could also lead to more targeted and effective therapeutic strategies for managing myocardial ischemia in diabetic patients, such as optimizing AA levels to prevent heart damage while avoiding exacerbating factors like ferroptosis.

In a recent study, Lv et al. report on the cardioprotective effects of arachidonic acid (AA) in diabetic models of myocardial ischemia, as documented in their paper “Protective role of arachidonic acid against diabetic myocardial ischemic injury: a translational study of pigs, rats, and humans“ [1]. Conducted across various species, this research demonstrates a compelling divergence from the traditional understanding of AA’s role in ischemic contexts, particularly highlighting its potential in mitigating myocardial damage. Intriguingly, the study proposes that AA can enhance mitochondrial function, reduce oxidative stress, and decrease cell death, which suggests a novel therapeutic application for AA in treating myocardial ischemia in diabetic conditions. However, the study does not delve into how underlying diabetic conditions might influence AA’s metabolic pathways, a critical factor in understanding its varying impacts on heart disease. Under the catalysis of different enzymes, arachidonic acid (AA) is converted into eicosanoids that promote inflammation, blood clotting, and vasoconstriction, and epoxyeicosatrienoic (EET), which have vasodilatory and anti-inflammatory effects. This dual nature of AA highlights its role as both a foe and a friend in ischemic heart disease, underscoring the critical importance of understanding and managing these opposing effects in addressing the condition (Fig. 1).

**Fig. 1:**
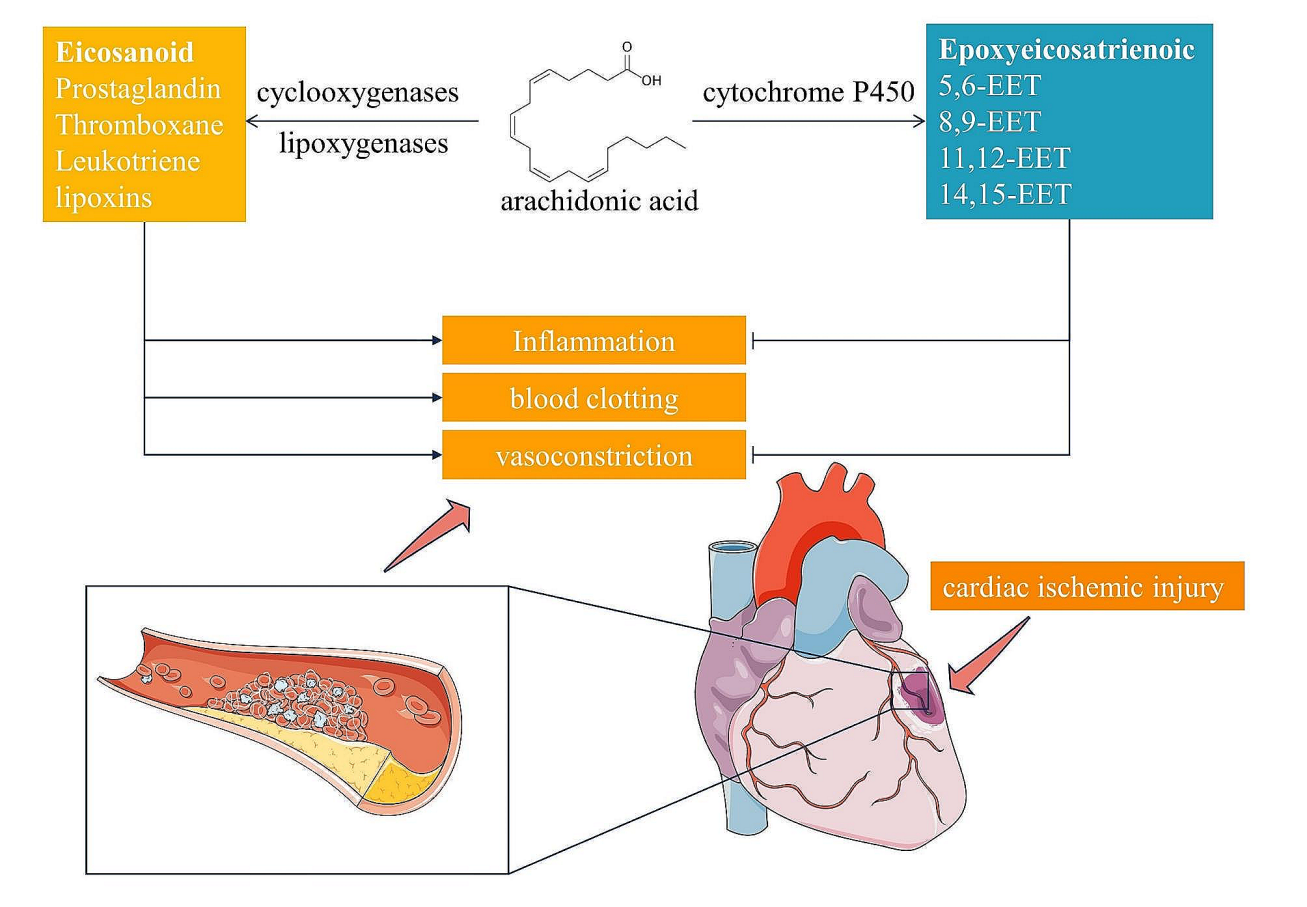
The dual role of arachidonic acid in ischemic heart disease

Despite the longstanding belief that the benefits of consuming polyunsaturated fatty acids outweigh the drawbacks, recent studies have challenged this perception [2]. Ferroptosis, a newly discovered form of cell death dependent on iron, has been identified as primarily caused by the accumulation of lipid peroxides, disturbances in iron metabolism, and imbalances in the amino acid antioxidant system [3]. Extensive research has demonstrated that ferroptosis not only plays a crucial role in the development and progression of significant chronic diseases but also assumes varied roles across different disease contexts [4]. In cardiovascular and neurodegenerative diseases, the occurrence of ferroptosis contributes directly to the pathogenesis, progression, and outcomes by causing damage to and loss of function in normal tissues and organs; but in the field of tumors, it is an important treatment method. Consequently, targeting ferroptosis could effectively prevent and slow the progression of these diseases [5].

AA, known for promoting the peroxidation of polyunsaturated fatty acids, has been implicated in facilitating ferroptosis, especially in ischemic heart diseases [6]. A 2022 study highlighted that acyl-CoA synthetase long-chain family member 4 (ACSL4) activates arachidonic acid into arachidonoyl-CoA, which is then esterified into phospholipids, suggesting that exogenous arachidonic acid can enhance RSL3-induced ferroptosis [7]. Recently, Brent Stockwell, often referred to as the father of ferroptosis, published another paper asserting that polyunsaturated fatty acids are key drivers behind ferroptosis [8].

Thus, the findings by Lv et al. suggesting a protective role for AA, appear to contradict the extensive evidence linking AA to pathways that exacerbate ischemic injury through ferroptosis. The potential reasons for these contradictory findings might lie in the specific diabetic context of the animal models used in the study. Diabetes could alter the typical metabolic pathways of AA, potentially through changes in enzyme activity or the levels of proteins involved in its metabolism, thereby affecting its role in the cell death process. While this hypothesis is compelling, it requires rigorous examination to determine the molecular mechanisms that could reverse AA’s role from a pro-ferroptotic agent to a protective agent in diabetic myocardial ischemia.

Additionally, considering that diabetic patients tend to develop iron overload over time, the primary reasons may include chronic inflammation increasing iron release, oxidative stress requiring iron to combat free radicals, insulin resistance altering iron metabolism, and non-alcoholic fatty liver disease affecting liver iron processing capabilities. Improper diet can also lead to increased iron intake. As one of the three main factors contributing to ferroptosis, disturbances in iron metabolism could exacerbate myocardial damage in diabetic patients more readily in practical scenarios. I must question whether the study has adequately addressed confounding factors, such as the impact of diabetes on iron metabolism and the specific conditions under which AA’s effects were evaluated [9].

Looking ahead, the study by Lv et al. reveals intriguing possibilities for using arachidonic acid (AA) therapeutically in diabetic myocardial ischemia. Despite conflicting with established views on AA’s role in ferroptosis, this opens up new research avenues. Understanding the specific effects of AA and its clinical implications is essential. Developing targeted therapies that harness AA’s cardioprotective attributes while mitigating its pro-ferroptotic effects could substantially improve treatments for ischemic heart disease in diabetic patients. I value the insights this study offers and anticipate further research to elucidate AA’s multifaceted role in cardiovascular health.

## Data Availability

No datasets were generated or analysed during the current study.
